# Neuronal Activity in the Rat Pulvinar Correlates with Multiple Higher-Order Cognitive Functions

**DOI:** 10.3390/vision4010015

**Published:** 2020-03-01

**Authors:** Fang-Chi Yang, Rebecca D. Burwell

**Affiliations:** 1 Cognitive, Linguistic & Psychological Sciences, Brown University, Providence, RI 02912, USA; fang-chi_yang@brown.edu; 2 Department of Neuroscience, Brown University, Providence, RI 02912, USA

**Keywords:** spatial, attention, egocentric, allocentric, reward

## Abstract

The pulvinar, also called the lateral posterior nucleus of the thalamus in rodents, is one of the higher-order thalamic relays and the main visual extrageniculate thalamic nucleus in rodents and primates. Although primate studies report the pulvinar is engaged under attentional demands, there are open questions about the detailed role of the pulvinar in visuospatial attention. The pulvinar provides the primary thalamic input to the posterior parietal cortex (PPC). Both the pulvinar and the PPC are known to be important for visuospatial attention. Our previous work showed that neuronal activity in the PPC correlated with multiple phases of a visuospatial attention (VSA) task, including onset of the visual stimuli, decision-making, task-relevant locations, and behavioral outcomes. Here, we hypothesized that the pulvinar, as the major thalamic input to the PPC, is involved in visuospatial attention as well as in other cognitive functions related to the processing of visual information. We recorded the neuronal activity of the pulvinar in rats during their performance on the VSA task. The task was designed to engage goal-directed, top–down attention as well as stimulus-driven, bottom–up attention. Rats monitored three possible locations for the brief appearance of a target stimulus. An approach to the correct target location was followed by a liquid reward. For analysis, each trial was divided into behavioral epochs demarcated by stimulus onset, selection behavior, and approach to reward. We found that neurons in the pulvinar signaled stimulus onset and selection behavior consistent with the interpretation that the pulvinar is engaged in both bottom–up and top–down visuospatial attention. Our results also suggested that pulvinar cells responded to allocentric and egocentric task-relevant locations.

## 1. Introduction

The thalamus in mammals can serve as a relay station for the transmission of information between cortical areas. Although an earlier view posited that thalamic nuclei passively relay information to the neocortex, recent studies suggest that the thalamus might play a role in actively modulating cortical areas via reciprocal connections [1,2,3,4,5]. Based on emerging anatomical and electrophysiological evidence, there are two types of thalamic relays [6,7,8]. The first-order thalamic relays include core nuclei, such as the lateral geniculate nucleus and the medial geniculate nucleus, receive primary afferents from the periphery (eyes and ears, respectively), and connect with sensory cortical areas. The higher-order thalamic relays include matrix nuclei, such as the pulvinar, and connect reciprocally with neocortical association areas [2,6]. 

In the primate brain, the pulvinar is the largest nucleus of the thalamus and is purportedly involved in visual attention [1,9]. Unilateral lesions of the pulvinar in patients are associated with spatial neglect and visual attention deficits [10,11]. Recent studies hypothesized that the pulvinar serves as a modulator for coordinating neuronal activity across multiple cortical areas involved in visual perception and attention [1,12,13,14]. Several monkey studies provided evidence for this view by showing that pulvinar activity modulates cortico-cortical interactions, including fronto-parietal and V4 with temporal occipital (TEO) interactions, when monkeys performed visual attention tasks [3,15,16]. More studies are needed to understand how thalamocortical interactions support visual attention.

The pulvinar in rodents, as well as its putative homolog in primates, receives inputs from the superior colliculus (SC) [17,18,19] and connects heavily with the visual cortex and the posterior parietal cortex (PPC) [17,20,21]. The role of the PPC in spatial attention is well established [22,23,24,25,26], but there are open questions about the contribution of its thalamic input from the pulvinar. In addition, although the pulvinar in the rat is thought to support attention [26], to our knowledge there is no electrophysiological evidence exploring the role of the rat pulvinar in visuospatial attention.

We previously examined the behavioral correlates of PPC cells in rats performing a visuospatial attention task (VSA). This task was adapted from the five-choice serial reaction time task [27] for use in our floor projection maze apparatus [28,29]. In the VSA task, rats were required to visually monitor multiple locations in space in order to make a correct response for a food reward. PPC activity showed a variety of correlates in the VSA task, including stimulus onset, spatial location of the target, target choice, and trial outcome [30]. Our prior findings provided evidence that the PPC engages top–down control in the translation of perception to action when visuospatial attention is engaged [30]. Because the PPC is strongly and reciprocally connected with the pulvinar in the rat [20,31,32] and the pulvinar is considered a higher-order thalamic relay nucleus, we hypothesized that the functions of the pulvinar go beyond relaying visual sensory information to include higher-order cognitive processes.

To address our hypothesis, we recorded neuronal activity in the rat pulvinar during performance on the VSA task. In this task, rats are required to use controlled attention at the beginning of the trial when monitoring multiple locations for the onset of a target stimulus. Stimulus-driven attention is engaged by the onset of the stimulus. Rats are then required to make a decision about the target location. Correct decisions are followed by a food reward. The VSA task thus engages visual attention and perception, decision-making, and reward learning. In addition, we used a version of the VSA task that allows dissociation of neuronal activity correlated with egocentric and allocentric reference frames.

## 2. Methods

### 2.1. Subjects

Subjects were five male Long–Evans rats (Charles Rivers Laboratories, Wilmington, MA) individually housed in a temperature-regulated colony maintained on a 12:12 h light:dark cycle. Experiments were carried out in the light phase. All procedures using animals were conducted in accordance with the Animal Welfare Act and were approved by the Brown University Animal Care and Use Committee (protocol #18-12-000, approved on 02/05/2019).

### 2.2. Apparatus

**Figure 1:**
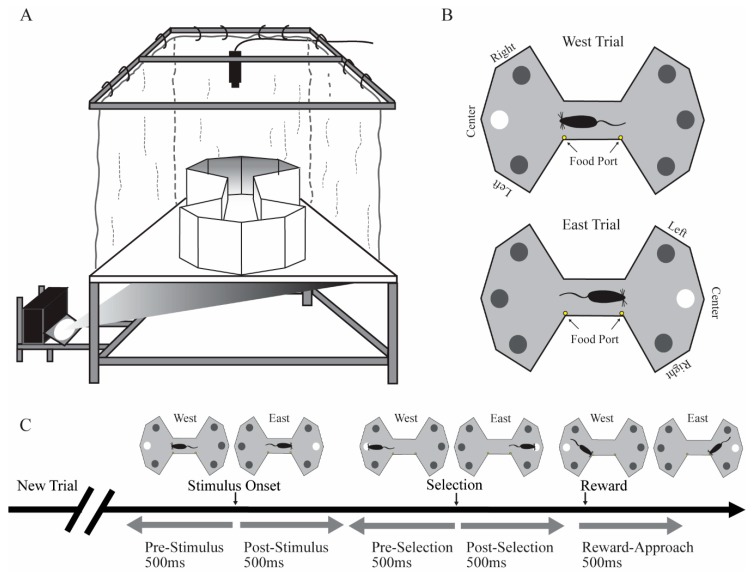
The visuospatial attention (VSA) task. (**A**) Schematic of the floor projection maze with the bowtie-shaped enclosure used for the task. (**B**) Top–down view of west vs. east trials. Trials were initiated when the rat stopped in the ready position (middle of the maze) and faced to one side of the maze (either west or east). After a variable period, the target location was briefly illuminated. The animal selected a target location by approaching one of the three locations on the same side, and then returned to the food port. A food reward was delivered following correct trials. After the animal consumed the reward, a new trial on the alternate side would be triggered immediately. (**C**) A single trial was divided into five behavioral epochs for analysis purposes. These five epochs included the following: the pre-stimulus and post-stimulus epochs were the 500 ms periods immediately before and after stimulus onset, respectively; the pre-selection and post-selection epochs were the 500 ms periods immediately before and after the rat selected a target by approaching the location, respectively; the reward-approach epoch was the first 500 ms after the animal entered the middle of the maze where the food ports were located to collect a food reward.

Rats were tested on the floor projection maze, an apparatus that exploits the natural tendency of rats to attend to items located on or close to the ground and that permits automated control over visual stimuli [28,29]. The floor projection maze is a horizontal rear projection screen, which serves as a floor to any shaped arena and allows back-projection of visual stimuli from underneath (Figure 1A). The apparatus has a clear Plexiglas subfloor (147.32 cm × 111.80 cm and 1.25 cm thick) covered by Dual Vision Fabric (Da-Lite Screen Company, Warsaw, IN), a unity gain flexible fabric designed for rear screen projection. A thin Plexiglas sheet (0.32 cm) covered the fabric for protection. Visual stimuli were projected onto the unity gain fabric from below the subfloor using an LCD projector (WT610 projector, NEC Corporation). In this experiment, the enclosure was a bowtie-shaped arena for the presentation of stimuli. Food reward (milk with various flavors) was delivered by two automated pumps (Med Associates, Inc., St. Albans, VT, USA) to stainless steel food ports located at the middle region of the maze. Auditory stimuli were controlled by an automated auditory stimulus generator (ANL926, Med Associates, Inc.) and delivered through a speaker located above the maze.

The floor projection maze was interfaced with three Windows PC systems, for location tracking, behavioral control, and neuronal data acquisition. Tracking was accomplished with a single camera using CinePlex Studio and Editor (v3.4.1) with Tracking and Basic Behavior modules (Plexon, Inc.). The position and body movements of the rat were recorded by calculating and tracking the centroid. Position data were analyzed online and saved in a data file for offline analysis, if needed. Based on the location of the rat, this system presented visual stimuli, collected behavioral data, and controlled delivery of reward. A Multichannel Acquisition Processor (MAP, Plexon Inc.) and SortClient (Plexon, Inc.) recorded real-time neuronal activity and behaviorally relevant event timestamps for later analysis. The MAP system was interfaced with the Med Associates system (DIG-713A SuperPort TTL Input Module and a DIG-726 SuperPort TTL Output module) used for controlling the projector, reward pumps, and audio signals.

### 2.3. Behavioral Training

Rats were put on food schedules to maintain body weight at 85%–90% of free feeding weight. After handling for at least 7 days, rats were habituated to the behavioral room for 10 min/day for three days. Rats were shaped and trained in the VSA task to a behavioral criterion prior to implantation. In the shaping sessions, rats were first trained in a 30 min session to approach a visual target stimulus for a food reward (a drop of flavored milk). In the initial shaping sessions, we adopted an errorless shaping procedure such that when the rat moved toward one of the three locations in one side of the maze, the visual stimulus at that location would illuminate and a tone would signal a correct choice. A new trial on the other side of the maze would be initiated after the rat entered the ready position of the other side of bowtie maze. After this initial shaping phase, rats were trained to stop in the ready position zone located in the middle of the bowtie shaped maze facing the side of the maze on which the target stimulus would be presented (Figure 1B). After a variable delay to wait for a stimulus presentation, a visual stimulus would illuminate in one of three randomly chosen locations. There was a short response window for rats to approach the location of the visual stimulus. Approach to the correct location was signaled by a brief tone and presentation of a drop of flavored milk as a food reward. If the rat approached an incorrect location, no reward was given, the trial was terminated, and a new trial would begin immediately. Two food ports were in the middle of the maze. One food port was closer to the east side of the maze that would provide a drop of flavored milk after the animal made correct selection in east trials. The other food port was closer to the west side of the maze that would offer a drop of flavored milk after a correct selection in the west trials. Rats were gradually trained in a series of steps culminating in the final parameters of the task. The duration for rats to stay in the ready position was gradually increased from 0.1 to 1.6 s. Visual stimulus duration was gradually decreased from 20 to 0.5 s. The response time window was gradually decreased from 20 to 5 s. In the final stage, rats were required to stay in the ready position for a variable pre-stimulus interval (1.2–1.6 s) until stimulus onset. The 0.5 s stimulus presentation was followed by a 5 s response time window.

The behavioral performance criterion was 70%–80% accuracy. Chance on the VSA task is 33.33%. Rats required 2–3 months of training to reach the behavioral criterion on the final stage of the task. After rats had reached criterion for 5 to 7 consecutive days, we conducted surgery to implant a hyperdrive for electrophysiological recording.

### 2.4. Surgery

Animals were premedicated with diazepam (2–5 mg/kg; i.p.), glycopyrrolate (0.05 mg/kg; s.c.), carprofen (5 mg/kg; s.c.), and butorphanol tartrate (0.5 mg/kg; s.c.) to counteract respiratory effects of anesthesia, to control pain, and to decrease risk of seizures. They were brought to a surgical level of anesthesia with isofluorane (1.0%–2.5%). Using a stereotaxic apparatus (Kopf, Tujunga, CA), rats were unilaterally implanted with a custom hyperdrive into the pulvinar at −3.9 mm AP, ± 1.8 mm ML, and −4.0 mm DV relative to bregma. Three rats were targeted in the left pulvinar; two rats were targeted in the right pulvinar. The hyperdrive had fifteen microdrives, each consisting of a drivable screw with guide tubing containing one stereotrode. Five microdrives of each hyperdrive were implanted in the pulvinar. Stereotrodes were made of two 12 μm twisted, formvar-insulated nichrome wires (A-M systems, Sequim, WA, USA). A full turn of the screw advanced the stereotrode by 350 μm. Two silver ground wires were wrapped around anchor screws in the skull. The hyperdrive was secured to the skull by the ground screws, small anchor screws, grip cement (Dentsply Caulk, Milford, DE, USA), and dental cement (Coltene/Whaledent Inc., Cuyahoga Falls, OH, USA).

### 2.5. Histology

After the last recording session, the rats were deeply anesthetized with an overdose of Beuthanasia-D (100 mg/kg, i.p.), and the final recording site was marked with an electrolytic lesion. The rats were then perfused with normal saline, followed by 4% formalin. The brains were post-fixed for 24 h in 4% formalin and then transferred to a 30% sucrose solution until sectioning. The brains were sectioned at 40 μm and stained for Nissl material with thionin.

### 2.6. Single-Neuron Recording

Neuronal activity recorded from stereotrodes, was amplified with a gain of 2 through a 31-channel wireless head stage (Triangle BioSystems Inc., Durham, NC, USA). Signals were passed through a high-gain amplifier (total gain = 10,000, MAP system, Plexon, Inc., Dallas, TX, USA). Single-unit activity was filtered between 0.8 and 6 Hz. The signal was then digitized at 40 kHz for single-unit activity. These signals were extracted through real-time thresholding (Sort Client, Plexon, Inc). The final waveforms were stored with timestamps of relevant events and position information for later analysis.

### 2.7. Single-Neuron Activity Analysis

Spikes associated with putative individual cells were isolated offline based on waveform characteristics and using a variety of partially automated and manual techniques (Offline Sorter, Plexon, Inc.). The result was a dataset for each cell containing timestamps corresponding to spike times and behaviorally relevant event markers. These datasets were further analyzed using Neuroexplorer (NEX, Nex Technologies, Madision, AL, USA), SPSS (IBM Corporation, Somers, NY, USA), and Matlab (Mathworks, Natick, MA, USA).

Firing rates for each cell were analyzed for behavioral correlates using two methods. The primary method was factorial analysis of variance (fANOVA), but we confirmed those findings with the bootstrapping approach described below. Firing rate was the dependent variable. For each cell, we first computed the mean firing rate (spikes/s) for each of five epochs on each trial. The five epochs included the following: the pre-stimulus and post-stimulus epochs were the 500 ms periods immediately before and after stimulus onset, respectively; the pre-selection and post-selection epochs were the 500 ms periods immediately before and after the rat selected a target by approaching the location, respectively. Lastly, the reward-approach epoch was the first 500 ms after the animal entered the middle of the maze to collect a food reward. The stimulus event was the onset of the target stimulus (500 ms illumination the possible target locations). The selection time event was the moment the rat entered a zone just in front of the location in which the target stimulus had appeared (Figure 1C). Entry of the ready position of the other side of bowtie maze triggered the next trial. Firing rate was the dependent variable for the fANOVAs. In the first set of analyses, the between-trial variable was outcome (correct response vs. incorrect response), and the two within-trial variables were stimulus onset (pre-stimulus vs. post-stimulus) and selection time (pre-selection vs. post-selection). The outcome was analyzed for the reward-approach epoch.

In a second set of analyses, we examined neural correlates associated with the location of the target stimulus. Based on the location of stimulus presentation, we pooled trials in which the target stimulus was at the same side of the maze (east vs. west) for analyzing allocentric location correlates. We then pooled trials in which the target stimulus was at the same egocentric location (left, right, and center) for analyzing egocentric location correlates. Only correct trials were used in this series of analyses. Thus, the between-trial variable was allocentric location (east vs. west) or egocentric location (left, right, and center). Sessions were analyzed for location only if there were at least three correct trials on each side. Allocentric location was analyzed separately for the post-stimulus, pre-selection, post-selection, and reward-approach epochs. The pre-stimulus epoch was not analyzed for trial location because there was no information about the allocentric location. Egocentric location was analyzed for the post-stimulus, preselection, and post-selection epochs without pre-stimulus and reward-approach epochs.

To confirm the results of the first series of analyses (fANOVAs), we used a bootstrapping procedure. For each cell in each recorded session, we randomly shuffled the firing rates for epochs analyzed across all trials 1000 times to create 1000 shuffled datasets. For example, if the reward approach epoch was the epoch under analysis and there were 100 trials, the 100 firing rates for the reward epoch were shuffled to create one new dataset, and this was done 1000 times. We then compared the original F value to the F values from the shuffled datasets. The cell was considered to be selective if the observed F value was higher than 95% of the distribution of the F values from the shuffled datasets.

The level of significance for all analyses was *p* < 0.05.

## 3. Results

### 3.1. Histology

**Figure 2:**
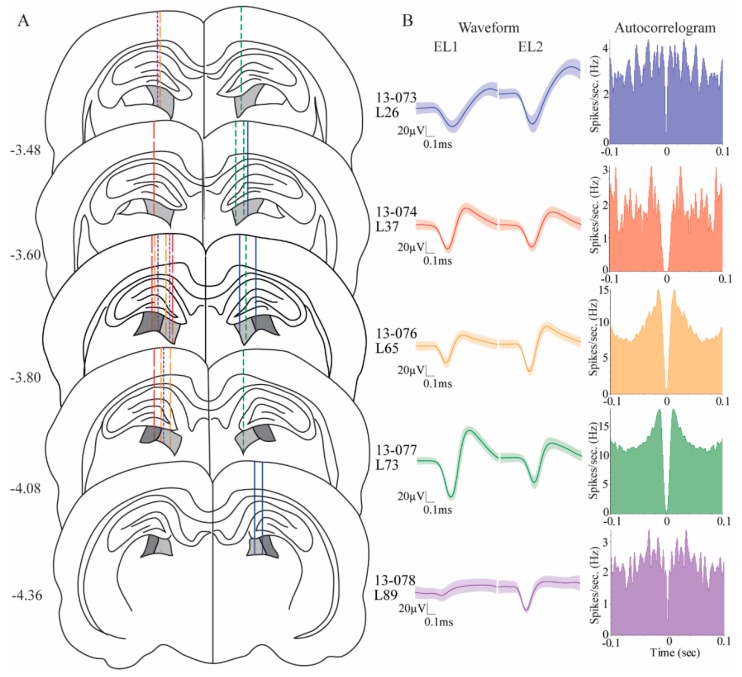
Location of stereotrodes and examples of isolated single units. (**A**) Histological locations of implanted stereotrodes. Target locations, the medial rostral pulvinar and lateral rostral pulvinar, are shown in light gray and dark gray, respectively. Five different line colors/patterns show stereotrodes from five animals (13-073, blue; 13-074, red; 13-076, orange; 13-077, green; 13-078, purple). The same colors were used to indicate stereotrodes from the five animals. (**B**) Five examples of isolated cells, one from each of five animals. The left panel shows waveforms (mean ± standard deviation, shaded) recorded from two electrodes (stereotrode) for each cell. EL1 and EL2 indicate the waveforms from individual electrodes of the same stereotrode. The right panel shows corresponding autocorrelograms for each cell. Bin width = 1 ms.

Examination of Nissl-stained coronal brain sections from each of the five animals indicated locations of all stereotrodes that were in the medial rostral pulvinar and lateral rostral pulvinar between ~3.5 and 4.4 mm posterior to the bregma and between 1.5 and 3.5 mm lateral to the midline (Figure 2A). Five different colors/weights of line were used to indicate stereotrodes from five animals (Blue solid line: Rat13-073; Red dash line: Rat13-074; Orange dash line: Rat13-076; Green dash line: Rat 13-077; Purple dash line: Rat13-078). Same color indicated stereotrodes from the same animal.

### 3.2. Pulvinar Cells Signaled Stimulus Onset and Predicted Behavioral Outcome

All five animals yielded isolated units. A total of 94 pulvinar cells that had good quality waveforms and were well clustered were used in the following analyses. Figure 2B shows five examples of isolated cells, one from each of the five animals. The left panel in Figure 2B shows waveforms (mean ± standard deviation, shaded) and the right panel shows the corresponding autocorrelogram. Each cell was recorded for a single session. The majority of the pulvinar cells, 74 (78.72%) exhibited behavioral correlate(s) during at least one of the task-relevant epochs as indicated by significant main effects or interactions of stimulus onset, target selection time, location, or outcome.

**Figure 3:**
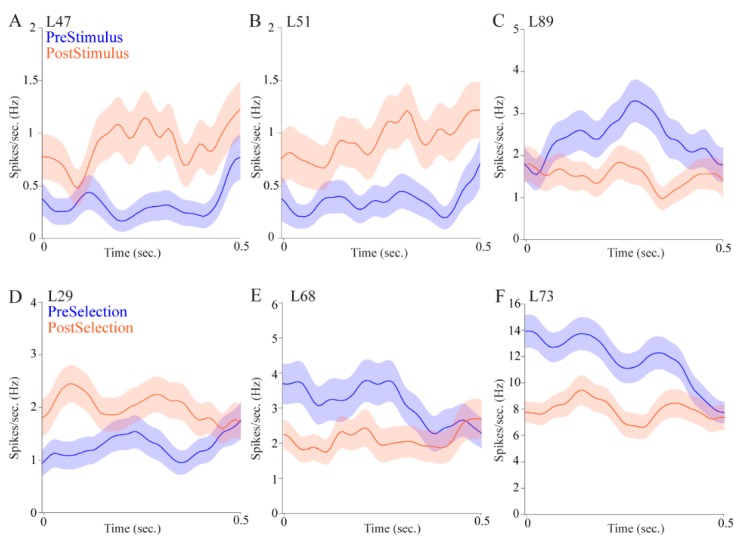
Pulvinar cells signaled attention to visual stimuli and target selection. (**A**–**C**) Mean firing rate (solid lines) and standard error (shaded) in pre-stimulus and post-stimulus epochs, shown in blue and orange, respectively. (**A**,**B**) Two example cells had significantly higher firing rates during post-stimulus epochs. (**C**) One example cell showed significantly higher firing rate during the pre-stimulus epoch. (**D**–**F**) Mean firing rates (solid lines) and standard error (shaded) of pre-selection and post-selection epochs, shown in blue and orange, respectively. (**D**) One example cell had a significantly higher firing rate during post-selection epochs. (**E**,**F**) Two example cells showed significantly higher firing rates during pre-selection epoch.

To investigate whether the pulvinar cells signaled attention to visual stimuli, we first analyzed neuronal responses to stimulus onset by comparing mean firing rates for the 500 ms before (pre-stimulus epoch) vs. 500 ms after the onset (post-stimulus epoch) of the 500 ms illumination of the target stimulus for correct vs. incorrect trials. Of all pulvinar cells, 27 (28.72%) cells showed some form of selectivity for stimulus onset. Of these cells, 18 cells (19.15%) showed significant increases or decreases in firing rate associated with attention to stimulus onset, alone (Table 1). For example, two pulvinar cells shown in Figure 3A,B fired significantly more during the post-stimulus epoch than the in the pre-stimulus epoch. Another pulvinar cell shown in Figure 3C had a significantly higher firing rate during pre-stimulus epoch. Five (5.32%) pulvinar cells fired differentially depending on whether the animal paid attention to make a correct selection when the stimulus came on. Four (4.26%) pulvinar cells exhibited a main effect of outcome alone. Thus, a total of 9 (9.57%) pulvinar cells fired differentially depending on the subsequent outcome of the trial (Outcome Only cells and Stimulus and Outcome cells in Table 1). Results for both the bootstrapping procedure and fANOVA were highly correlated (Pearson *r* = 0.99, *p* < 0.00001, Table 1).

### 3.3. Pulvinar Cells Signaled Target Selection and Behavioral Outcome

To understand whether the activity of pulvinar cells correlated with target selection, we analyzed neuronal responses to animals’ selection behavior by comparing mean firing rates for the 500 ms before (pre-selection epoch) vs. 500 ms after the selection (post-selection epoch) for correct vs. incorrect trials. We observed that seventeen (18.09%) pulvinar cells signaled selection by firing significantly differently across the pre-selection and post-selection epochs (Table 1 and Figure 3D–F). Figure 3D shows an example cell that had significantly higher firing rates during the post-selection epoch. The other two selective cells responded significantly more during the pre-selection epoch (Figure 3E,F).

We also found that nine (9.57%) pulvinar cells signaled both selection and outcome and seven (7.45%) pulvinar cells responded to outcome alone during the peri-selection interval. Thus, 26 (27.66%) pulvinar cells differentiated target selection (Selection only cells and Selection and Outcome cells in Table 1). A total of 16 (17.02%) pulvinar cells displayed differential firing patterns related to behavioral outcome (Outcome Only cells and Selection and Outcome cells in Table 1).

**Figure 4:**
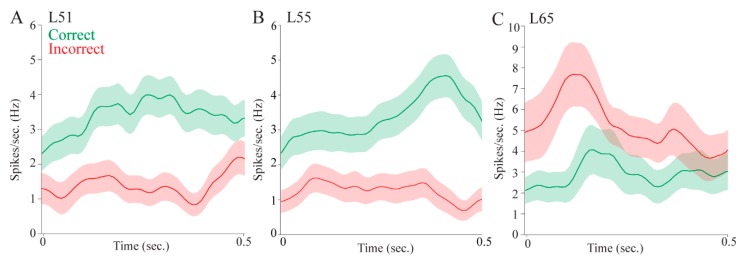
Pulvinar cells distinguished behavioral outcomes. Mean firing rates (solid lines) and standard error (shaded) of correct and incorrect outcomes during reward-approach epoch, shown in green and red, respectively. (**A**,**B**) Two example cells had significantly higher firing rates in correct trials. (**C**) An example cell significantly increased firing rates in incorrect trials.

For the reward-approach epoch, in which the animal had just entered the food area after selecting a target location, 19 (20.21%) pulvinar cells displayed selectivity for outcome (Table 1). Three representative reward-selective cells are shown in Figure 4. Two example cells fired significantly more in correct trials than incorrect trials (Figure 4A,B); one example cell fired more in incorrect trials (Figure 4C).

### 3.4. Pulvinar Cells Signaled Allocentric and Egocentric Location

We previously reported that PPC cells displayed selectivity for the location of the target stimulus, as demonstrated by differential firing patterns on left vs. right trials [30]. In the present study, we had the opportunity to examine the spatial property of its thalamic associate, the pulvinar, in the processing of allocentric and egocentric information. We analyzed the post-stimulus, pre-selection, and post-selection epochs for correct trials because spatial location was relevant during these epochs and well-controlled on correct trials. The reward-approach epoch was also analyzed for allocentric location correlates. As expected, some level of location selectivity was evident in all analyzed epochs (Table 1). Based on the location of stimulus presentation, we pooled trials in which the target stimulus was in the same side of the maze (east vs. west) for analyzing allocentric location correlates. We then pooled trials in which the target stimulus was at the same egocentric location (left, right, and center) for analyzing egocentric location correlates.

**Figure 5:**
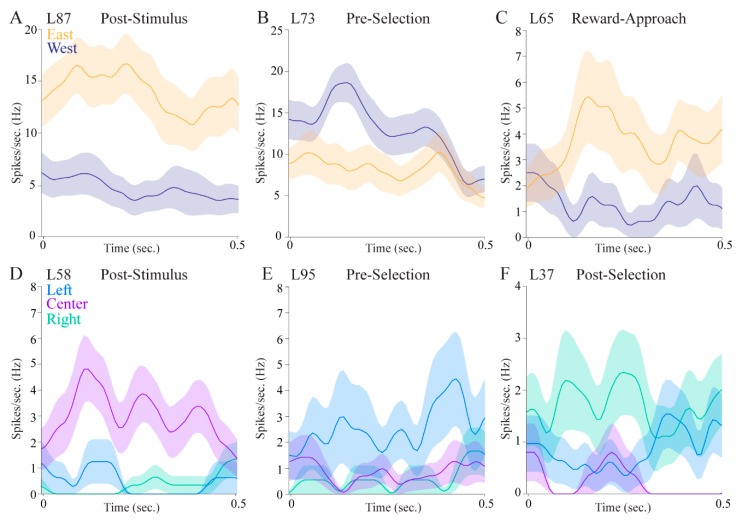
Pulvinar cells distinguished allocentric locations (east vs. west) and egocentric locations (left vs. center vs. right) in various behavioral epochs. For allocentric analyses, mean firing rates (solid lines) and standard error (shaded) of east and west trials are shown in orange and dark blue, respectively. For egocentric analyses, mean firing rates (solid lines) and standard error (shaded) of left, center, and right trials are shown in light blue, light purple, and green, respectively. (**A**) An example cell with significantly higher firing rates in east trials during the post-stimulus epoch. (**B**) An example cell with significantly higher firing rates in west trials during the pre-selection epoch. (**C**) An example cell with significantly higher firing rates in east trials when the animal approached the reward area. (**D**) An example cell with significantly higher firing rates in center trials during the post-stimulus epoch. (**E**) An example cell with significantly higher firing rates in left trials during the pre-selection epoch. (**F**) An example cell with significantly higher firing rates in right trials after the animal made a selection.

Regarding allocentric location correlates, we observed significant differences in east vs. west trials for 18 (19.15%) pulvinar cells in the in the post-stimulus epoch, 13 (13.83%) cells in the pre-selection epoch, four (4.26%) cells in the post-selection epoch, and 13 (13.83%) cells in the reward-approach epoch, a total of 35 (37.23%). Eight of these cells responded differentially in the east vs. west side of the maze in more than one epoch. Of those eight cells, five cells exhibited higher firing rates on the same side of the maze across epochs. Figure 5A–C shows three example cells that distinguished different allocentric locations in each analyzed epoch (post-stimulus, pre-selection, and reward-approach, respectively).

Regarding egocentric location correlates, we observed significant differences in firing rates among left, center, and right trials in the pulvinar for eight (8.51%) cells in the post-stimulus epoch, five (5.32%) cells in the pre-selection epoch, and 18 (19.15%) cells in the post-selection epoch. A total of 28 (29.79%) pulvinar cells differentiated left, center, and right trials in at least one behavioral epoch. Three example cells shown in Figure 5D,E had significantly higher firing rates only in preferred egocentric locations in the analyzed epoch. Firing rates showed slight fluctuations over time suggesting that the pulvinar cells might respond to the real-time changes of egocentric locations relative to the animals’ movement.

Among all selective cells responding to either allocentric or egocentric location, 14 cells showed both allocentric and egocentric selectivity during at least one task-relevant epoch of the VSA task. Thus, 49 out of 94 cells (52.13%) exhibited task-relevant location selectivity while animals performed the VSA task.

Finally, in a series of post hoc analyses, we examined the question of whether there were differences in selectivity across the two pulvinar subregions in which our electrodes were located, the medial rostral pulvinar and lateral rostral pulvinar. Cells in the rostral lateral pulvinar had significantly higher proportions of egocentric cells (42.11%), compared to the cells in the rostral medial pulvinar (21.43%, *χ*^2_(1)_^ = 4.63, *p* = 0.03). There were no differences between these two areas in numbers of cells with allocentric selectivity (37.50% in rostral medial pulvinar; 36.84% in rostral lateral pulvinar; *χ*^2_(1)_^ = 0.004, *p* = 0.95). Cellular correlates of stimulus-driven attention and target selection behavior did not differ across these subregions.

## 4. Discussion

Using the VSA task, we previously reported that neuronal correlates in the PPC signal stimulus onset, target selection, and outcome, suggesting a role in multiple cognitive functions during visuospatial attention [30]. In the present study, we used a similar VSA task to study the function of the rat pulvinar, the major thalamic input of the PPC. Our double-sided version of the VSA task allowed the presentation of multiple stimuli on two sides of the maze permitting identification of both egocentric and allocentric spatial correlates. We report that pulvinar cells show selectivity for stimulus onset, choice, and outcome. In addition, more than half of the cells exhibited some type of task-relevant location selectivity. These findings support our hypothesis that the pulvinar is engaged in multiple, high-level cognitive functions, including both bottom–up and top–down attention, and that the pulvinar shows correlates of multiple spatial frames of reference. To our knowledge, this is the first study to examine neuronal correlates of pulvinar cells in a visuospatial attention task in rats.

Regarding attention, pulvinar cells fired differentially in the pre- and post- stimulus epochs. This could mean that pulvinar cells signaled stimulus onset, showing that the pulvinar is involved in stimulus-driven attention. Some cells, however, fired more during the pre-stimulus interval, which could be a correlate of controlled attention as the rat is monitoring the possible target locations. Other cells fired more during the post-stimulus interval which, as we have suggested, could be a correlate of stimulus onset. Pulvinar cells also signaled target selection and reward, providing further evidence for a role in reward-guided decision-making.

Over a third of the cells displayed allocentric location selectivity and nearly a third showed egocentric location selectivity, indicating that the pulvinar is involved in processing information about different spatial reference frames. Some cells had both allocentric and egocentric selectivity, providing further evidence that the pulvinar is involved in translating spatial correlates across different frames of reference. Interestingly, the magnitude of the firing rate differences appears to be larger for allocentric cells compared with egocentric cells (Figure 5, compare upper panels to lower panels). This may have to do with the particular epochs in which cells tended to show location correlates. Allocentric cells were more likely to show selectivity in the post-stimulus epoch immediately after stimulus onset, when the rat was in the center of the maze facing the fan-shaped part of the maze where stimuli appear on either the east or the west side. In contrast, egocentric cells were more likely to be selective in the epoch after a choice was made, when the animal was physically located in the left, center, or right in one or the other of the fan-shaped parts of the maze. This suggests that allocentric cells may be more sensitive to visual information, whereas egocentric cells may be more sensitive to spatial information. Alternatively, the egocentric cells could show lesser differences across conditions because they are more likely to show mixed selectivity given that three locations are analyzed. Indeed, the cell shown in Figure 5F fires more to the right and left locations than to the center locations.

Our evidence that the rodent pulvinar is engaged under visuospatial attentional demands and signals task-relevant locations is consistent with a recent monkey study reporting that neuronal activity in the pulvinar, similar to the parietal cortex, significantly increased when a target appeared at the cued location indicating spatial correlates to behaviorally relevant locations [3]. Thus, similar to the monkey pulvinar, the rat pulvinar can be considered a subcortical hub that supports visuospatial attention [12,14].

The pulvinar in both primates and rats is widely considered part of a visual attention circuit [1,26,33,34], but there have been disagreements about homology across rodents and primates. Two recent papers reviewed the evidence that the pulvinar is homologous across species [19,35]. An examination of nine species of rodents, tree shrews, and primates suggests that at least some subregions of the pulvinar are homologous across species based on molecular markers and connectional similarities, particularly connectivity with the SC, visual cortex, and visual association areas [35]. In the rat, the pulvinar is divided into the medial rostral pulvinar, the medial caudal pulvinar, and the lateral pulvinar based on cytoarchitecture. The medial rostral pulvinar receives heavy inputs from the visual cortex and almost no SC inputs. The medial caudal pulvinar receives the strongest projections from the SC and almost no inputs from the visual cortex. The lateral pulvinar is further subdivided into the rostral and caudal portion based on its connectivity with the SC. The rostral portion of the lateral pulvinar receives weak SC projections and heavy projections from the visual cortex, whereas the caudal portion of the lateral pulvinar receives input from the superficial layers of the ipsilateral SC and weak projections from the visual cortex [17]. 

Our electrodes were located in the medial rostral and lateral rostral pulvinar (Figure 2). These two subareas of the pulvinar both receive heavy inputs from the visual cortex and weak projections from the SC [17]. Cellular correlates of stimulus-driven attention and target selection behavior did not differ across these subregions. We did, however, observe that cells in the rostral lateral pulvinar had significantly higher proportions of egocentric cells compared to the cells in the rostral medial pulvinar. There were no differences between these two areas in proportions of allocentric cells. Our findings are in line with the notion that the medial pulvinar may be more involved in ventral visual stream functions and the lateral pulvinar may be more involved in dorsal visual stream functions, as has been suggested for the primate brain [36]. Future research is needed to understand how these subregional differences in the rat pulvinar map onto subregional differences in the primate pulvinar.

To summarize, the present findings were aimed at understanding the function of thalamocortical connections in visuospatial attention. We previously reported neuronal correlates of the PPC in multiple cognitive processes when rats performed a VSA task [30]. Here, we provided the first evidence that the rat pulvinar—the major thalamic input to the PPC—is engaged in tasks that have visuospatial attentional demands. We further demonstrated that the pulvinar is involved in multiple cognitive functions, as might be expected from a higher-order thalamic relay. These functions include top–down and bottom–up attention, decision-making, and processing of information about spatial reference frames. The present work, together with our previous findings, implicates both the PPC and pulvinar in the multiple cognitive processes tapped by the VSA task. This research lays the groundwork for understanding the role of thalamocortical interactions in visuospatial attention.

## Figures and Tables

**Table 1:** Numbers and percentage of selective cells.

Epoch (Criterion Cells)	Correlate	Pulvinar Cells (%) ANOVA	Pulvinar Cells (%) Bootstrapping
Peri-Stimulus	Stimulus Only	18 (19.15)	18 (19.15)
	Stimulus and Outcome	5 (5.32)	5 (5.32)
	Outcome Only	4 (4.26)	4 (4.26)
	Total	27 (28.72)	27 (28.72)
Peri-Selection	Selection Only	18 (19.15)	17 (18.09)
	Selection and Outcome	8 (8.51)	9 (9.57)
	Outcome Only	6 (6.38)	7 (7.45)
	Total	32 (34.04)	33 (35.11)
Reward-Approach	Outcome	19 (20.21)	19 (20.21)
Post-stimulus	Allo-Location	19 (20.21)	18 (19.15)
Pre-selection	Allo-Location	14 (14.89)	13 (13.83)
Post-selection	Allo-Location	4 (4.26)	4 (4.26)
Reward-Approach	Allo-Location	13 (13.83)	13 (13.83)
	Total	37 (39.36)	35 (37.23)
Post-stimulus	Ego-Location	8 (8.51)	8 (8.51)
Pre-selection	Ego-Location	5 (5.32)	5 (5.32)
Post-selection	Ego-Location	19 (20.21)	18 (19.15)
	Total	29 (30.85)	28 (29.79)
Total Selective Cells		70 (74.47)	74 (78.72%)

Shown are numbers of the criterion cells for each analysis and numbers/percentages of cells that displayed significant main effect or interactions during selective behaviorally relevant epochs. A total of 94 pulvinar cells were included in all analyses. Results from both the original fANOVA and the bootstrapping procedure are shown. For fANOVA analyses, the significant level was *p* < 0.05. For the bootstrapping procedure, the 95th percentile was the cutoff for selectivity. Results for the two sets of analyses were highly correlated (Pearson *r* = 0.99, *p* < 0.00001).
